# Remote Control of Excited-State Chemistry in Positive Reversibly Switchable Fluorescent Proteins

**DOI:** 10.1021/acs.jpclett.6c02808

**Published:** 2026-10-20

**Authors:** Anam Fatima, Danielle Rosenberger, YongLe He, James N. Iuliano, Gregory M. Greetham, Partha Malakar, Andras Lukacs, Peter J. Tonge, Stephen R. Meech

**Affiliations:** † School of Chemistry, 6106 University of East Anglia, Norwich NR4 7TJ, U.K.; ‡ Department of Chemistry, 12301 Stony Brook University, Stony Brook, New York 11794, United States; § Central Laser Facility, Research Complex at Harwell, 97008 Rutherford Appleton Laboratory, Didcot OX11 0QX, U.K.; ∥ Department of Biophysics, Medical School, 37656 University of Pecs, 7624 Pecs, Hungary

## Abstract

Positive reversibly switchable fluorescent proteins (+rsFPs) are genetically encoded fluorescent labels allowing super-resolution microscopy without the fading inherent in the better established negative (−) rsFPs. Typically, switching is initiated by an ultrafast excited-state isomerization reaction. Here, we investigate the ultrafast photophysics of four structurally similar variants of the archetypal +rsFP Padron, including Padron0.9, Padron2, Kohinoor, and a newly reported “reverse” Kohinoor mutant (Kohinoor S176F) using ultrafast optical and vibrational spectroscopy. Padron0.9 exhibits an efficient excited-state proton transfer (ESPT) reaction in parallel with photoisomerization, whereas Kohinoor and Padron2 do not. Remarkably, introducing the single reverse mutation S176F in Kohinoor restores Padron-like ESPT dynamics, even though the mutation is remote from the proton donor. This result demonstrates that a single amino acid residue near to, but not in direct H-bonded interaction with, the chromophore can govern the fate of excited states. TRIR measurements reveal distinct changes in the local hydrogen-bonding network and chromophore–protein interactions associated with ESPT-active variants and further reveal a key role for H-bonding between the chromophore and an arginine residue through a study of the R66K mutant of Padron0.9. Overall, these results show that subtle mutations shift the excited state between proton transfer and photoisomerization pathways, providing mechanistic insight into photoswitching in rsFPs, thus guiding the design of improved fluorescent proteins for superresolution bioimaging.

## Graphical abstract

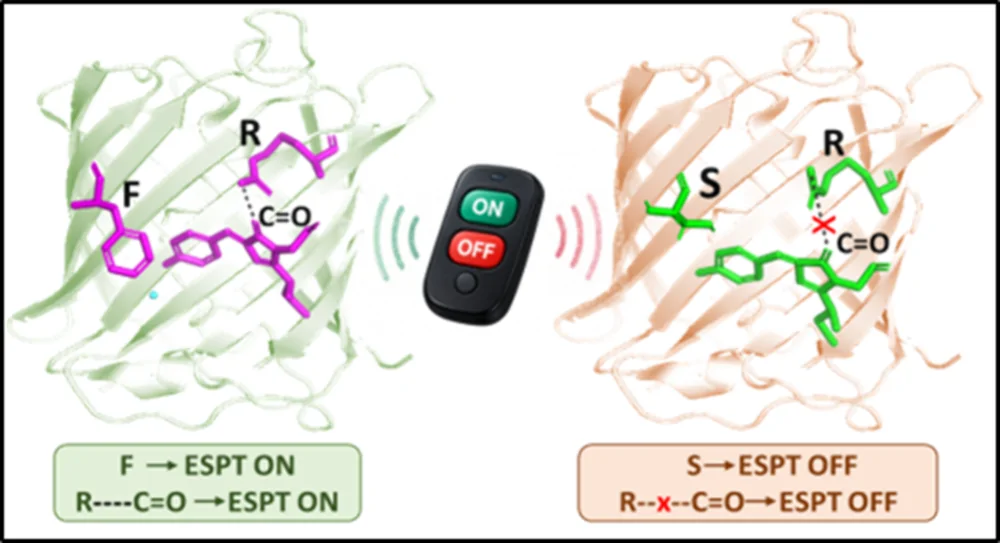

Reversibly switchable fluorescent proteins (rsFPs) have emerged as important tools in advanced bioimaging, particularly in super-resolution microscopy,^1,2^ where their ability to toggle between emissive (bright/on) and nonemissive (dark/off) states underpins sub diffraction limited spatial resolution.^3^ Their switching mechanism is typically driven by cis–trans photoisomerization of the chromophore, often coupled to changes in protonation state.^4–6^ In negative (−) rsFPs such as Dronpa, the on-state corresponds to a deprotonated cis-chromophore and the off-state to a protonated trans-chromophore.^5,7,8^ Positive (+) rsFPs, of which Padron is the archetype, however, operate differently: the off-state consists of a deprotonated trans-chromophore, while the on-state exists as an equilibrium of protonated and deprotonated cis forms.^6,9^ Excitation of the protonated cis drives switching to the off-state, whereas excitation of the deprotonated form produces fluorescence and maintains the protein in the on-state. It has been proposed that +rsFPs are particularly attractive for super-resolution microscopy, as their switching scheme avoids the loss of fluorescence during data acquisition, a limitation encountered with −rsFPs.^10^

Different variants of +rsFP (Padron0.9, Padron2 and Kohinoor) have been characterized in terms of their switching performance, including switching kinetics, fatigue resistance, and brightness.^11,12^ Intriguingly, the three variants differ only by a small number of mutations, mostly in residues which are distant from the chromophore. Padron0.9 is derived from Padron and differs from it by only two mutations both distant from the chromophore,^6^ whereas Kohinoor and Padron2 derived from Padron0.9 carry seven and eleven mutations, respectively of which only one in Kohinoor and two in Padron2 are located near the chromophore.^10,12^

Despite the largely remote mutations, these proteins differ significantly in both their photostability and switching efficiencies.^11,12^ Further, ultrafast spectroscopic studies reveal different photophysical pathways in the variants. Fron et al. examined the ultrafast dynamics of Padron and reported that the excited neutral cis-chromophore (*on* state, A*) undergoes rapid decay with a ca. 1 ps time constant, likely corresponding to an excited-state proton transfer (ESPT), followed by isomerization on a ca.14 ps time scale.^9^ A study of Padron0.9 using ultrafast transient absorption (TA) in the UV–visible range by Walter et al. confirmed ESPT as the initial reaction step following excitation to A*, leading to formation of the emissive anion, I*.^13^ However, direct observation of a subsequent cis–trans isomerization was not achieved, which the authors attributed to the low switching quantum yield (ca. 2%).

Recently, we reported the on- to off-switching mechanism in Kohinoor, a Padron variant with higher on–off switching quantum yield (8%).^14^ Surprisingly, we did not observe ESPT in Kohinoor, despite there being only subtle differences between its crystal structure and that of Padron0.9 (Figure 1a). Rather excited-state isomerization was the dominant relaxation mechanism. This observation raised the question: how do these subtle structural differences give rise to such distinct excited-state pathways? Single-site mutations are known to alter the spectral properties and excited-state pathways of fluorescent proteins, as in E222Q GFP, where the E → Q substitution rewires the proton transfer pathway leading to efficient photochromism or in Dronpa where the M159T mutation enhances on-to-off switching yield.^15,16^ Comparison of the crystal structures of Padron0.9 (3LS3) and Kohinoor (9DVE) points to only a single significant difference in the immediate chromophore environment. Padron0.9 contains a phenylalanine residue at position 173, whereas Kohinoor carries a serine at the equivalent position (S176, Figure 1a). We hypothesized that the F173S substitution may have led to suppression of ESPT in Kohinoor by altering the local chromophore environment. The interaction map (generated using LigPlot+,^17^ Figure S1) of the on-state of Padron0.9 shows that the surrounding protein residues including E140 and I195 stabilize a water molecule near the phenolic group of the chromophore. This water molecule is absent in Kohinoor (Figure S1). We speculated that this change may be related to the Phe → Ser replacement which appears to alter the local environment in a manner that destabilizes the water molecule seen in Padron0.9. We proposed that in Kohinoor, the smaller S176 permits a rearrangement of Y162 bringing S145 closer to the chromophore phenol (2.45 Å, see Figure S1), reducing the space available for the water molecule. In contrast, substitution of Ser by the bulkier Phe residue restricts the local rearrangement, maintaining S145 at a greater distance from the chromophore phenol, thereby allowing a water molecule to be positioned near the phenol of the chromophore. Here, we test that hypothesis by introducing the “reverse” mutation (S176F) into Kohinoor. Remarkably, this single mutation substantially restores *Padron-like* behavior, as shown by the observation of ESPT. The finding supports the idea that this residue, although not directly interacting with the chromophore, is nevertheless a critical determinant of its excited-state dynamics.

**Figure 1:**
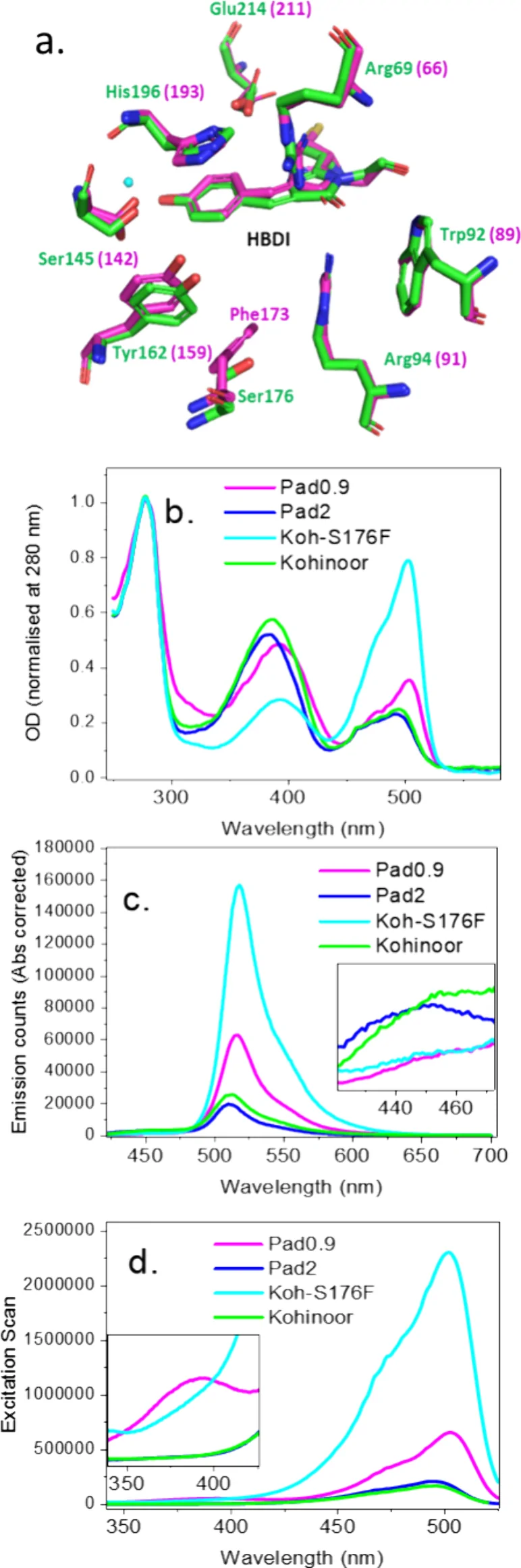
(a) Structural overlay of the on-states of Kohinoor (PDB: 9DVE, green) and Padron0.9 (PDB: 3LS3, pink) showing key residues around the chromophore. The blue sphere represents the water molecule present in Padron0.9 but not resolved in Kohinoor. (b) Ground-state absorption spectra of the four protein variants recorded in D_2_O at pD 8, normalized to the Trp absorption at 280 nm. (c) Absorption-corrected emission spectra excited at 410 nm showing the dominant emission band at ca. 510 nm. The inset highlights a broad emission feature around 450 nm observed in Padron2 and Kohinoor but absent in Padron0.9 and Koh-S176F. (d) Excitation spectra monitored at 530 nm emission. The inset shows a distinct excitation band near 380 nm in Padron0.9, indicating contribution of the neutral chromophore to the anionic emission, in Koh-S176S there is a positive signal but no clear maximum, whereas this feature is absent in Kohinoor and Padron2.

To further establish the role of this mutation, we also investigated Padron2, which independently carries the same Phe → Ser substitution (F173S) found in Kohinoor. Although a crystal structure is not available for Padron2, the F173S mutation is one of only two mutations (R66K and F173S) directly surrounding the chromophore, and therefore most likely to influence the ESPT dynamics. It should be noted that both Padron0.9 and Kohinoor retain the native arginine residue (R69 in Padron0.9 and R66 in Kohinoor) as in avGFP,^18^ whereas Padron2 replaces it with lysine. If this residue participates directly in proton transfer, the R66K substitution could potentially alter the ESPT pathway. However, previous work by Walter et al. on Padron0.9 and our results on Kohinoor show that Padron0.9 exhibits ESPT whereas Kohinoor does not, despite both retaining the R66 residue. This observation suggests that the R66K mutation is unlikely, on its own, to be the determining factor controlling ESPT activity. Thus, on sequence comparison alone, S176F substitution in Kohinoor appears to be the critical mutation controlling the rate and dynamics of ESPT. In this work, we revisit Padron0.9 and investigate Koh-S176F and Padron2 using fluorescence upconversion to isolate the ESPT pathway, UV–visible TA to characterize other dynamic pathways, and transient infrared (TRIR) spectroscopy to probe the interaction of chromophore and protein. TRIR measurements reveal a role for H-bonding to the chromophore carbonyl in ESPT, which we probe through the R66K mutation. We demonstrate that single mutations reshape the excited-state landscape and modulate the photophysics of rsFPs.

The steady-state absorption spectra of Padron0.9, Padron2, Kohinoor, and Koh-S176F recorded in D_2_O (pD 8) exhibit two main bands: one at 390 nm, corresponding to the cis neutral (A) and another at 500 nm, attributed to the cis anionic form (B) (Figure 1b). The 500 nm absorption band is most intense in Koh-S176F suggesting that the mutation shifts the protonation equilibrium toward the anionic form of the chromophore [although there is no direct interaction between the residue F and the phenol of the chromophore (Figure 1a and Figure S1), as discussed above].

Excitation to B* at 500 nm produces strong emission at ca. 510 nm (see absorption corrected emission spectra in Figure 1c) in all four protein variants. Upon excitation to A* at 410 nm, Padron2 and Kohinoor show dual emission with weak A* emission at 460 nm and strong I* emission at 510 nm (I* and B* differ in the arrangement of surrounding residues, as originally described for avGFP^19^), whereas Padron0.9 and Koh-S173F exhibit predominantly a single emission band at 510 nm with only very weak emission near 460 nm. The excitation spectrum of Padron0.9 monitored at 510 nm emission shows a weak band around 400 nm along with the expected dominant band at 500 nm, suggesting that a part of the 510 nm emission arises from excitation of A* via ESPT. The excitation spectrum of Koh-S176F, does not show a discernible peak due to A*, probably due to the larger contribution from direct excitation of the large underlying B state population, which thus absorbs more strongly at 400 nm than in Padron0.9 (Figure 1b). However, there is a positive contribution below 400 nm. In contrast, the excitation spectrum of Padron2 at 510 nm shows that the anionic emission arises exclusively from the direct excitation of B* with no detectable signal at around 400 nm, similar to what is observed in Kohinoor.^14^ Therefore, the excitation signal around 400 nm already suggests a role for ESPT from A*, although partially masked in Koh-S176F. The emission is consistent with this picture, with quenching of the 460 nm A* emission in Padron0.9 and Koh-S176F (Figure 1c) potentially due to fast ESPT in these proteins.

Significantly, both the absorption and emission spectra of Padron0.9 and Koh-S176F are ca. 10 nm red-shifted compared to Kohinoor and Padron2. A 20 nm red-shift was reported for H148D GFP mutants which was attributed to a very short H-bond between the chromophore phenol and the Asp148 residue, or to electrostatic interactions introduced by the negatively charged residue.^20,21^ The observation of a red-shift in Padron0.9 and Koh-S176F may then arise from a stronger H-bond between S145 and the phenol/phenolate (which have the distances 2.4 Å in Padron0.9 vs 2.7 Å in Kohinoor).

To build on these steady-state observations, we used ultrafast time-resolved fluorescence (TRF).^22^ In principle, TRF directly resolves ESPT through a rise in the I* emission, reflecting its formation after excitation of A*, an established signature of ESPT in GFP.^19,23^ TRF measurements of Padron0.9, Koh-S176F and Kohinoor were performed by exciting A* at 400 nm and detecting the upconverted fluorescence at 510 nm (Figure 2). The experiments were carried out in both H_2_O and D_2_O, to resolve any H/D isotope effects on ESPT. The data in D_2_O are shown in Figure S2 and the time constants and the corresponding amplitudes in both H_2_O and D_2_O are summarized in Table S1. In Padron0.9, the signal exhibits a clear rise component, reflecting formation of I* via ESPT. Fitting the data with a sum-of-two exponentials model reveals an initial rise time of ca. 2 ps in H_2_O and ca. 4 ps in D_2_O at 510 nm, consistent with slower ESPT in the deuterated solvent. A larger isotope dependence was reported for the ESPT in GFP.^19^

**Figure 2:**
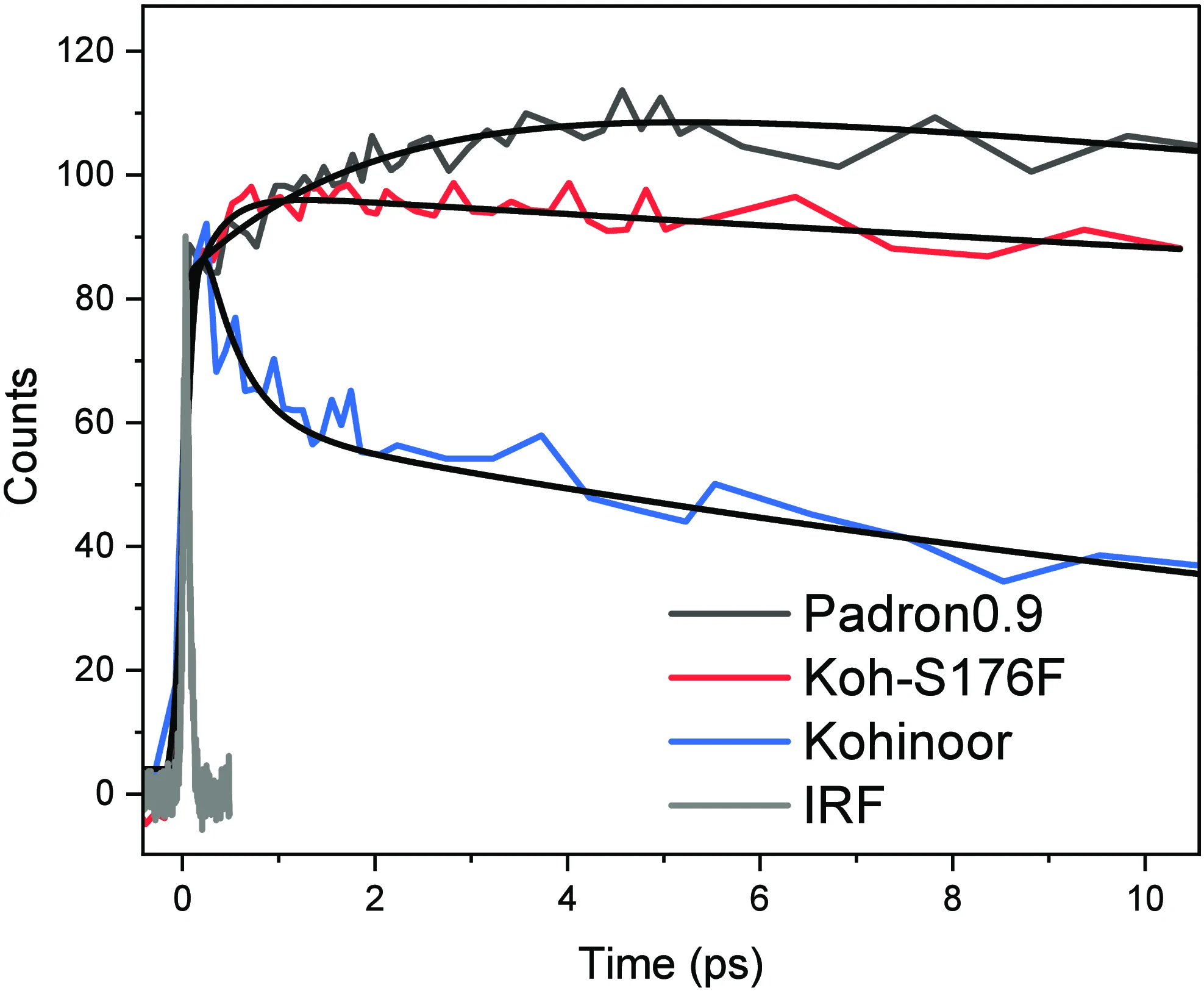
Time-resolved fluorescence upconversion data recorded in H_2_O under 505 nm LED irradiation to retain the cisH population, and measured at 510 nm (I* emission). Data are fit with a biexponential function. Kohinoor shows a fast decay component, whereas Padron0.9 exhibits a clear rise time, with an intermediate flattened signal in Koh-S176F. The trace in gray represents the instrument response function (IRF of ∼80 fs).

In contrast, Kohinoor shows only an ultrafast decay with no detectable rise component (Figure 2), indicating the absence of ESPT, in agreement with the steady-state results (Figure 1b). The fast decay is assigned to the contribution of A* emission which underlies B*.^24^ In the case of Koh-S176F, the 510 nm signal appears flattened rather than showing the distinct rise seen in Padron0.9, but unlike Kohinoor, does not exhibit a fast decay component. The intermediate behavior of Koh-S176F can be explained by the rising component due to ESPT being offset by the underlying fast decay of the A* state. In addition, a larger portion of B will be directly excited in Koh-S176F compared with Padron0.9, because of its larger population (Figure 1b).

Further detail for the on to off reaction in the three proteins was obtained by UV–Vis TA and TRIR experiments. In both cases the A state was excited with 100 fs pulses at 405 nm and probed either from 340 to 670 nm with 100 fs time resolution for TA or with 200 fs resolution in the 1350–1800 cm^–1^ range for TRIR. TA measurements were performed in both H_2_O and D_2_O, whereas TRIR measurements were conducted only in D_2_O buffer at pD8. Measurements were made under continuous-flow conditions, with the sample reservoir illuminated by a 505 nm LED to maintain the on-state population.

The TA spectra of Padron0.9, Koh-S176F and Padron2 at selected pump–probe delays are shown in Figure 3. As is evident from Figure 3a and b, the TA spectra of Padron0.9 and Koh-S176F are very similar and dominated by three principal features: a negative ground-state bleach (GSB) centered at ca. 380 nm, a negative stimulated emission (SE) beyond ca. 470 nm, and a positive excited-state absorption (ESA) band centered near 440 nm. At the earliest pump–probe delay shown (150 fs) the SE is a broad largely featureless negative band assigned to A* which decays within 10 ps to the baseline above ca. 600 nm, while a spectrally narrower feature grows in at 500 nm. The latter is consistent with SE from the I* species formed by ESPT, as was observed by Walter et al. in Padron0.9.^13^ This 500 nm feature then decays over the ps-ns time scale and does not fully recover the baseline within our 3 ns delay range. In parallel, the ESA at *ca*. 440 nm decays while the GSB at ca. 380 nm fills to be replaced by a weak excited-state absorption at 430 nm. The corresponding data in H_2_O show a faster ESPT reaction (SI, Figure S3). In contrast, the TA spectra of Padron2 show that the SE of the A* decays in picoseconds without any observable growth of a negative feature near 500 nm, and the SE fully returns the baseline within 2.5 ns, accompanied by decay of the ESA and recovery of the GSB. These observations in Padron2 are broadly consistent with our previous study on Kohinoor where no rise of the I* SE band was detected.^14^ Similarly, the TA data (Figure S4) on Padron0.9­(R66K) mutant do not show any rise in the I* SE band near 500 nm, indicating R → K substitution suppresses ESPT in Padron0.9, suggesting a role for the R66–chromophore C=O H-bond (see below).

**Figure 3:**
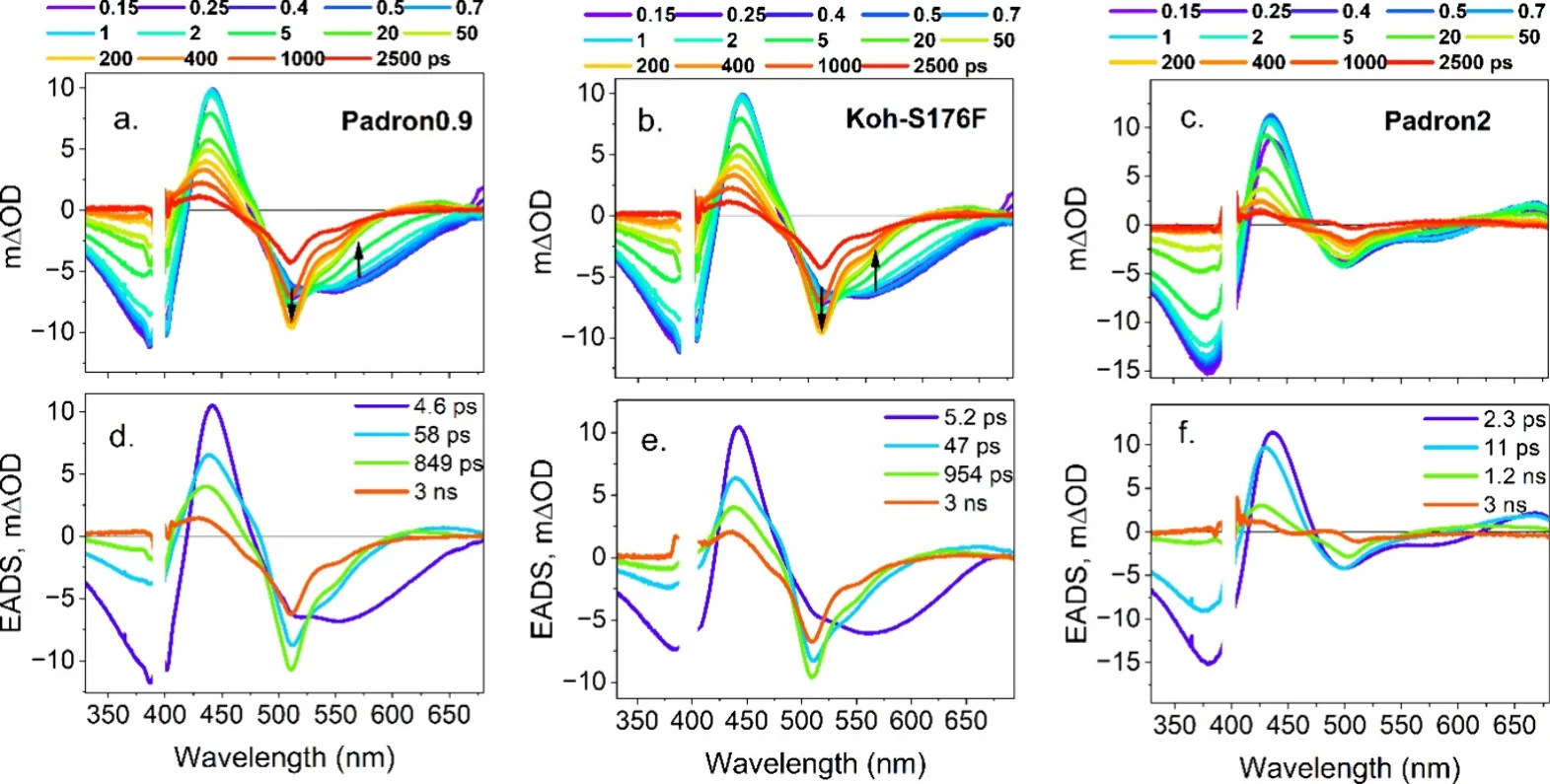
Transient absorption spectra of (a) Padron0.9, (b) Koh-S176F, and (c) Padron2 following 405 nm excitation of the cisH chromophore in D_2_O. The black arrows in panels (a) and (b) indicate the decay of SE from cisH accompanied by the growth of SE from I* formed via ESPT. (d, e, and f) Corresponding EADS obtained from global analysis of the TA data using a sequential model consisting of three first-order steps and a fixed long-time decay constant.

Global analysis of the TA data of Padron0.9, required a three-step kinetic model, yielding three characteristic time constants of 4.6, 58, and 849 ps plus a long-lived constant component set to 3 ns as shown in evolution associated difference spectra (EADS, Figure 3d.). Similar lifetimes are obtained from the global analysis of Koh-S176F data (see EADS, Figure 3e). The quality of fit for both proteins at key wavelengths and the corresponding decay associated difference spectra (DADS) are presented in the SI (SI Figure S5 and S6, respectively). In both cases, the first EADS is assigned to relaxation of the broad SE feature around 570 nm, and the ESA and GSB at 450 and 405 nm, respectively; these are associated with A* excitation. The main feature of the second EADS formed in ca. 5 ps is the shift and narrowing of the SE to 500 nm, associated with the formation of I*. This is consistent with ESPT, and in good agreement with the rise time of 4 ps resolved in TRF (Table S1, Figure S2). Essentially the same features are seen for Koh-S176S, confirming ESPT in that protein. These EADS convert to a third in 58 ps in Padron0.9 and 47 ps in Koh-S176F characterized by a further modest increase in the SE amplitude and a narrowing of the red edge in the SE at ca. 550 nm. This component most likely reflects a fraction of excited-state population undergoing ESPT on a slower time scale, suggesting an inhomogeneous distribution of the ground-state population in both proteins. The relaxation in the SE is accompanied by a recovery of the ground-state bleach and decay of the TA at 425 nm. This state then relaxes to a final EADS (itself assigned a 3 ns lifetime) in hundreds of ps, while retaining the I* SE profile. Thus, there is an overall nonsingle exponential relaxation of I* formed in ESPT possibly overlapped with decay of directly excited emission from a ground-state anion population (B*) underlying the A-state absorption. These distinct anion states need not decay on the same time scale, as they will have distinct protein environments thus explaining the nonsingle exponential relaxation, as was discussed in GFP itself.^19^ A similar underlying component was also observed in Kohinoor.^14^ Thus, the biexponential decay of the anion observed in both Padron0.9 and Koh-S176F, suggests contributions from two distinct emissive species: the I* state and the directly excited cis anion, B*.

Global analysis of TA data in H_2_O also yields four characteristic time constants (EADS are shown in Figure S3 and fits of the kinetic traces are in Figure S7). The components associated with ESPT are approximately two times faster than in D_2_O (for example: 2 ps in H_2_O vs 4.6 ps in D_2_O for Padron0.9) and are in good agreement with the TRF (2 ps in H_2_O vs 3.6 ps in D_2_O, Table S1). This is consistent with the ESPT reaction.

The EADS for Padron2 in D_2_O, (Figure 3f, DADS in SI Figure 6c), exhibit a biexponential monotonic decay (2.3 and 11 ps) of the A* SE, along with recovery of the GSB, and without any observable rise time in the SE associated with the anionic state. These lifetimes are therefore assigned to the on- to off-state photoisomerization reaction. The residual SE at 520 nm observed after 11 ps can be attributed to the directly excited cis anion, as was also seen in Kohinoor (ref 14 and SI Figure 7a), which largely returns to the baseline within 1.2 ns. Indeed, the first two excited-state lifetimes of 2.3 and 11 ps (Figure 3f) are quite comparable to those observed previously in Kohinoor (3 and 22 ps).^14^ Both Padron2 and Kohinoor carry the F173S substitution and their similar spectroscopic behavior strongly suggests that the presence of serine instead of phenylalanine plays a critical role in suppressing ESPT, and promoting the photoisomerization pathway.

Significantly, Padron0.9­(R66K) despite carrying the F residue, does not show ESPT, suggesting that R66 is also required for ESPT. Comparison of the crystal structures of Padron0.9 and Kohinoor (Figure S8) shows that R66 in Kohinoor is displaced away from the chromophore carbonyl which precludes the H-bond interaction present in Padron0.9. The relatively weaker H-bond (5.8 Å, Figure S8) in the former likely promotes photoisomerization, which suppresses the competing ESPT pathway. The H-bonding interactions between R66 and chromophore carbonyl are known to influence the local chromophore environment in Kaede type FPs.^25^ In one example, R66 H-bonds to the chromophore C=O, whereas the A69T mutant promotes R66-T69 interaction, altering the local H-bonding and electrostatic network. In that case, the altered environment restricts chromophore twisting and reduces the photoisomerization quantum yield. In contrast, in Padron0.9 and the Koh-S176F mutant (both having R66), substitution of serine with phenylalanine reopens the ESPT pathway, which occurs in parallel with the excited-state isomerization, the latter pathway being confirmed by the observation of ground-state recovery in the earliest EADS (Figure 3a,b). Together, these observations suggest that both R66 and F176 are required for ESPT (see Table 1) and play an interconnected role in determining the fate of the excited state. It is noteworthy that these residues lie far apart in the chromophore pocket and only R66 has a direct interaction with the chromophore. It is remarkable that these residues act cooperatively to support ESPT without either of them directly interacting with the phenol ring.

**Table 1:**
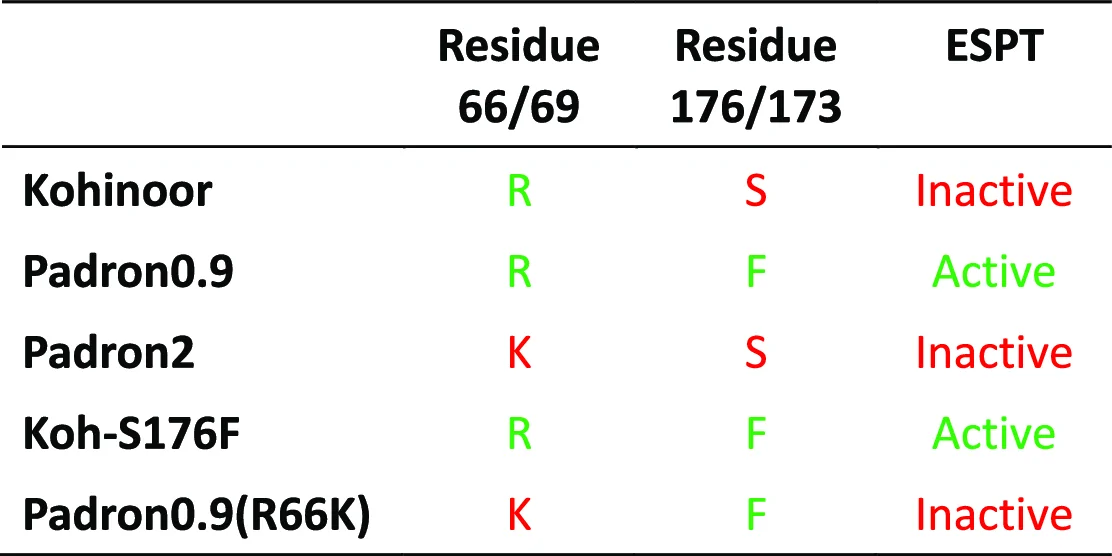
Padron Variants Studied and the Resulting ESPT Activity Showing Variants Carrying Both F and R Promote ESPT

Transient vibrational spectroscopy provides further insight into ESPT and interactions between the chromophore and its environment. The TRIR spectra of Padron0.9 and Koh-S176F excited at 405 nm are similar (Figure 4a and b) to one another but markedly different from those of Padron2 (Figure 4c), Kohinoor (ref 14. and Figure s9b) and Padron0.9­(R66K) (Figure S10). This indicates closely related chromophore environments in the ESPT-active variants. All five proteins exhibit bleaches characteristic of vibrational modes of the neutral GFP chromophore, already reported for avGFP.^26^ Specifically, in Padron0.9, the four prominent ground-state bleach features are at 1688, 1642, 1598, and 1514 cm^–1^ (Figure 4a), in Koh-S176F at ca. 1681, 1636, 1594, and 1512 cm^–1^ (Figure 4c), in Padron2 at 1680, 1636, 1594, and 1512 cm^–1^ (Figure 4e) and in Padron0.9­(R66K) at ca. 1684, 1645, 1597, and 1512 cm^–1^ (Figure S10). These also align with modes observed in Kohinoor (Figure S7b).^14^ An additional prompt bleach signal at ca. 1560 cm^–1^ in Padron0.9, 1555 cm^–1^ in Koh-S176F and 1570 cm^–1^ in Padron2, is not seen in the chromophore itself and is thus assigned to a vibrational mode arising from chromophore–protein interaction perturbed upon electronic excitation. Several transient absorption (positive) features are also observed in all five proteins (see Figure 4a-c, Figure S6b and Figure S10). In particular, and as in Kohinoor,^14^ the TRIR spectra show transient bands at ca. 1471, 1585, and 1627 cm^–1^ in Padron0.9, ca. 1471, 1582, and 1625 cm^–1^ in Koh-S176F, ca. 1473, 1580, and 1624 cm^–1^ in Padron2 and ca. 1475, 1583, and 1628 cm^–1^ in Padron0.9­(R66K), all appearing immediately following excitation. These features are assigned to specific protein modes coupled to the chromophore excitation through hydrogen bonds or other electrostatic interactions.^14^

**Figure 4:**
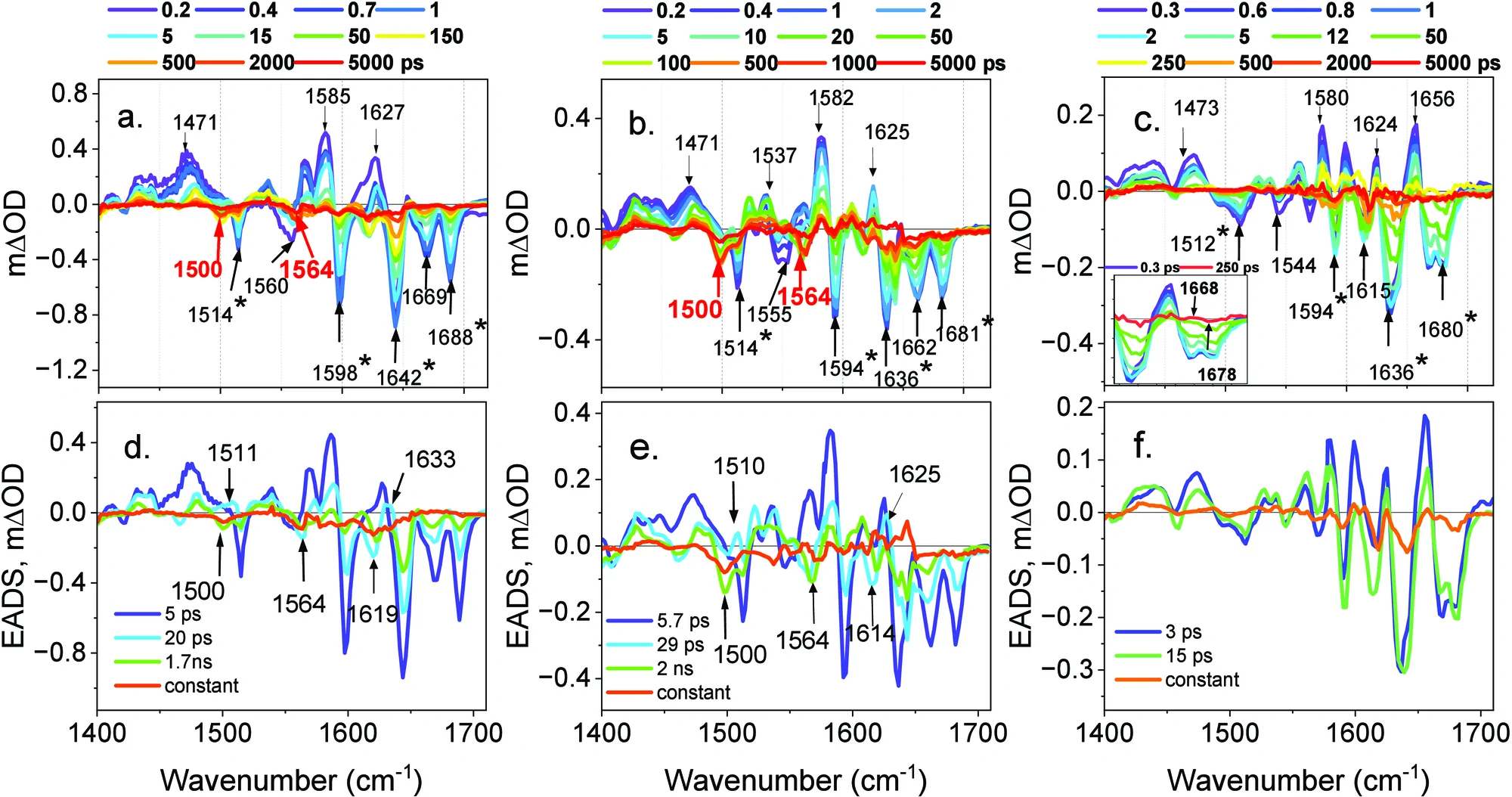
Time-resolved infrared spectra of (a) Padron0.9, (b) Koh-S176F, and (c) Padron2 following 405 nm excitation of the on-state neutral cis chromophore in D_2_O. The red arrows in panels a and b denote the appearance of bleaches after tens of picosecond only seen in the ESPT active rsFPs. (d, e, and f) EADS obtained from global analysis of the TRIR data using a sequential model consisting of three exponential decay components and a fixed long constant. Inset in panel (c) shows a small transient/bleach pair signal at ca. 1668/1678 cm^–1^ assigned to neutral trans photoisomer.

The TRIR spectra reveal significant difference in the GSB features between non-ESPT and ESPT-active proteins. In Kohinoor, a single bleach band is observed at ca.1680 cm^–1^ (see Figure S7b for Kohinoor spectra). This feature is assigned to the imidazolinone C=O stretching vibration of the chromophore and is seen on A* excitation in most FPs.^27^ In contrast, Padron0.9 exhibits an additional low-frequency bleach band at ca. 1669 cm^–1^ alongside the 1688 cm^–1^ feature. Both bleach bands exhibit identical kinetics, suggesting they both originate from the chromophore C=O rather than from distinct residues. The lower frequency C=O bleach band therefore likely reflects a different local environment experienced by a subpopulation of the chromophore imidazolinone ring. Interaction maps of Padron0.9 and Kohinoor (LigPlots in Figure S1) provide a plausible structural basis for this observation. In both proteins, the conserved arginine residue (R66) forms a hydrogen bond with the C=O of the imidazolinone. We suggest that at room temperature the two bleaches originate from chromophore subpopulations that differ in their interaction strength with R66, with the lower frequency bleach corresponding to a population which has the stronger H-bonding interaction. Koh-S176F exhibits the same pair of bleach bands as in Padron0.9, with the lower frequency bleach at 1662 cm^–1^. This is again consistent with a strengthened interaction between R66 and the chromophore C=O, at least in this subpopulation. The absence of this low-frequency mode in Kohinoor suggests a lack of hydrogen bonding between the chromophore carbonyl and R66, which is supported by the interaction maps (see LigPlot diagrams in the SI Figure S1). In Padron2, the earliest-time bleach at ca. 1680 cm^–1^ exhibits a bifurcation but with a much smaller splitting (11 cm^–1^) than that in Padron0.9 and S176F, suggesting weaker hydrogen bonding between the chromophore and the R66 residue. We speculate that the stronger H-bond interaction enhances the photoacidity of the chromophore in the two ESPT supporting proteins. Further, the ESPT-inactive Padron0.9­(R66K) mutant (see Figure S10), also displays a single carbonyl bleach centered at around 1684 cm^–1^, confirming that R → K substitution results in the loss of the H-bond with the chromophore carbonyl. Thus, the specific chromophore – R66 H-bond is required for ESPT, but it may be switched on/off by the presence/absence of the remote F176 residue.

There are two other notable differences between the TRIR of the ESPT and non-ESPT competent pairs. Within a few tens of ps of excitation both Padron0.9 and the Koh-S176F mutant show the appearance of two bleach features at ca. 1500 and ca. 1564 cm^–1^. This pair is more prominent in the Koh-S173F than in Padron0.9 but is absent in the non-ESPT active Kohinoor, Padron2 and Padron0.9­(R66K) (Figure 4e,f, SI Figure S9b, and Figure S10). In Padron0.9 and Koh-S176F, these transient signals decay on the same time scale as the recovery of the ground-state bleach, and that recovery remains incomplete within the 5 ns time window. Thus, we associate these signals with formation of I* by ESPT, although their assignment to a specific proton-accepting residue is not straightforward.

Finally, instantaneous formation of a transient band near 1656 cm^–1^ in the non-ESPT active proteins (e.g., Kohinoor and Padron2), is absent in Padron0.9 and Koh-S176F. This mode is also absent in the spectra of the isolated chromophore and is therefore assigned to a coupled chromophore–protein vibrational mode, presumably involving C=O stretching vibrations of the amide I backbone,^28^ perturbed on electronic excitation. The absence of this transient in the ESPT active proteins suggests that their chromophore–protein coupling differs from those in Kohinoor, Padron2 and Padron0.9­(R66K). Although the definitive assignment of this mode is not straightforward, its sensitivity to relatively small mutations in the chromophore pocket is notable and results in significant changes in the vibrational spectral features

Finally, in the non-ESPT-active variant Padron2, small spectral perturbations appear after a few hundred picoseconds, most notably the emergence of a transient/bleach pair at 1668/1678 cm^–1^ (see inset in Figure 4c), which we assign to formation of the neutral trans photoisomer, consistent with our previous assignment in Kohinoor.^14^ In contrast, Padron0.9 and Koh-S176F do not show comparable long-lived spectral features, consistent with lower photoswitching QY of Padron0.9 (2%) compared to Kohinoor (8%).^12,13^

The sequential three-step decay model for TRIR of both Padron0.9 and Koh-S176F (Figure 4b,d) converged with time constants of ca. 5 ps, 20 ps and 2 ns. The quality of the global fit is shown at representative frequencies in Figure S11 and the corresponding DADS are given in Figure S12a,b. The first EADS of 5 ps is consistent with the 4.6 ps component from TA and 4 ps rise time obtained in TRF associated with formation of I* by ESPT (Figure 3d and e, and Figure 2). This EADS is characterized by decay of the ESA bands and recovery of the four chromophore GSB modes mentioned above. This component gives rise to the second EADS showing transient signals at ca. 1511 cm^–1^ and a transient/bleach pair at ca. 1633/1619 cm^–1^ in Padron0.9 (Figure 3d) and 1510 cm^–1^ and 1625/1614 cm^–1^ in Koh-S176F; Figure 4e; these are thus assigned to ESPT marker bands. The third EADS appearing in few tens of ps, shows the formation of bleaches at 1500 and 1566 cm^–1^, presumably also associated with changes in the proteins induced by the ESPT reaction (see Figure 4d and e). The fourth EADS shows relaxation of these bands in ca. 1–2 ns to form a final constant difference spectrum.

The final EADS spectrum, appearing as a long-lived constant component in the global fit, indicates a persistent photoproduct arising from I* formation and associated changes in protein structure. There are no obvious modes that can be assigned to the deprotonated form (trans anion) ground-state species (e.g a mode at 1500 cm^–1^), probably because of the extremely low switching quantum yield (∼2%). The EADS of Padron2, gives two time components of ca. 3 and 15 ps (DADS shown in Figure S9c), consistent with those obtained in the TA (2 and 11 ps) and in close agreement with the Kohinoor.^14^ Therefore, these are assigned to subpopulations undergoing photoisomerisation at different rates, both leading to a long-lived photoproduct.

Finally, we turn to the assignment of the mutation effects. The role of R66 is revealed in TRIR as the formation of a specific H-bond interaction in a sub population of the ground state with the adjacent chromophore, red-shifting the C=O mode. This interaction likely stabilizes the quinoidal form of the chromophore through both H-bond and favorable electrostatic interaction, promoting ESPT. The role of the remote S176F in promoting ESPT is less clear as it is not interacting with the chromophore. The interaction maps (Figure S1) for Padron0.9 show a water molecule stabilized near the phenol of the chromophore through three residues (E140, S142 and I195). Thus, a possible assignment for the 1500 cm^–1^ bleach could be perturbation or loss of O–D bending modes associated with the trapped D_2_O molecule during ESPT. However, an unambiguous assignment requires further isotopic or computational analysis. The 1566 cm^–1^ bleach could potentially be assigned to protonation of E140 leading to loss of the carboxylate asymmetric stretch vibration. However, protonation of Glu should be accompanied by formation of a positive transient associated with the carbonyl stretch at ca. 1710 cm^–1^, as reported in avGFP.^29^ The absence of this feature in the present data precludes a definitive assignment to protonation of E140. Indeed, the 1566 cm^–1^ bleach may originate from a different protonation site or changes in the amide I backbone (β-sheet) upon ESPT.

The recovery of ESPT in Koh-S176F is consistent with a rearrangement of the chromophore pocket that permits a water molecule to be positioned adjacent to the chromophore phenol (Figure 1 and Figure S1). Although the presence of this water molecule could not be assigned unambiguously from the structure (Figure 1a), the recovery of the ESPT, evidenced by the growth of I* SE band in the TA spectra (Figure 2c) and several ESPT marker bands in the TRIR data, closely resembles the dynamics observed in Padron0.9, where the water is unambiguously observed. We therefore propose that S → F substitution in both Padron0.9 and Koh-S176F restores ESPT by maintaining the water-mediated proton relay. It is intriguing that the S176F substitution which lies far from R66 is linked to the movement of R66 closer to the chromophore carbonyl in Padron0.9 promoting ESPT but how the effect of mutation propagates across the chromophore pocket is unclear from the present data.

Overall, in the ESPT active variants (Padron0.9 and Koh-S176F), a sub population with R66 H-bonded to the chromophore C=O has ESPT occurring in competition with excited-state relaxation via internal conversion, which occurs on a few to tens of ps time scale. A small fraction of the internal conversion reactions lead to isomerization. In contrast, in Padron2 and Kohinoor, there is no competing ESPT pathway, and the excited-state population partitions between internal conversion and photoisomerization. Because the ESPT relaxation channel in the former pair competes with photoisomerization this provides a plausible explanation for the significantly lower on-to-off switching quantum yield of Padron0.9 (ca. 2%) compared to non-ESPT variants Kohinoor (ca. 8%) and Padron2 (ca. 11%) (Figure 5).^12,13^

**Figure 5:**
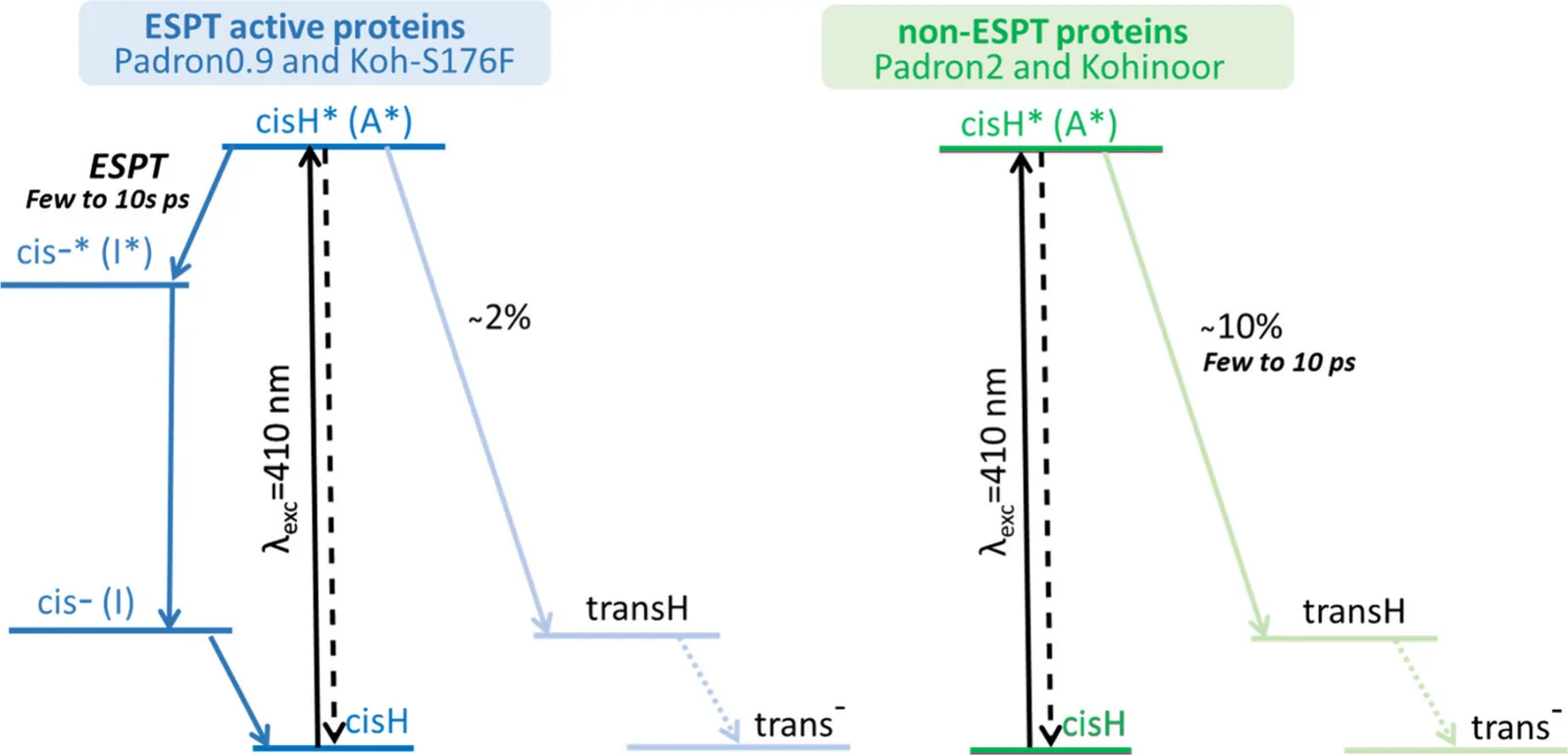
Comparison of excited-state relaxation pathways in ESPT active protein pair (Padron0.9 and Koh-S176F) with the non-ESPT pair (Padron2 and Kohinoor). In Padron0.9 and the “reverse” mutant Koh-S176F, S176F mutation switches on the ESPT (denoted by I*), which competes with photoisomerization, leading to a yield of ∼2%, In contrast, no ESPT was detected in the latter pair, promoting photoisomerization (∼10%).

In summary, our combined steady-state and time-resolved data demonstrate that a single mutation remote from the chromophore phenol (S176F) modulates the ESPT dynamics in +rsFPs. Padron0.9 and Koh-S176F exhibit closely related photophysics, characterized by ESPT from the neutral cis chromophore to form the anionic emissive state in competition with photoisomerization and internal conversion. In contrast, Kohinoor, Padron2 and Padron0.9­(R66K) show no evidence of ESPT and instead undergo picosecond time scale excited-state decay along an isomerization coordinate. Although definitive signatures associated with an ESPT proton acceptor could not be assigned from the present TRIR data, it was shown that the Phe → Ser and Arg → Lys mutations reorganize the local hydrogen-bonding network, particularly interactions between the conserved arginine near the imidazolinone carbonyl and a perturbed amide I mode in the excited state, producing distinct vibrational signatures. Such reorganization could modify the photoacid properties of the chromophore. Further, we suggest that the S176F mutation allows stabilization of a transient water molecule near the chromophore phenol to act as a proton acceptor. These findings establish a direct mechanistic link between single-site mutations, chromophore–protein interactions, and the balance between proton transfer and photoisomerization in +rsFPs, providing design principles for engineering fluorescent proteins with tailored switching properties for advanced imaging applications.

## Supplementary Material



## Notes

Additional experimental procedures for spectroscopy and sample preparation; further protein structure data; additional transient fluorescence, absorption, and TRIR data; additional data analysis including fitting quality (PDF)
