# Supplementary material for: Remote Control of Excited-State Chemistry in Positive Reversibly Switchable Fluorescent Proteins
Source: J Phys Chem Lett. 2026 Oct 20;17(43):12339–47. doi: 10.1021/acs.jpclett.6c02808 (PMC13726473; doi:10.1021/acs.jpclett.6c02808)
Supplement: Supplementary file 1 [file jz6c02808_si_001.pdf]

# Remote Control of Excited State Chemistry in Positive Reversibly Switchable Fluorescent Proteins

Anam Fatima,[1] Danielle Rosenberger,[2] YongLe He,[2] James N. Iuliano,[2] Gregory M. Greetham,[3] Partha Malakar,[3] Andras Lukacs,[4]* Peter J. Tonge[2]* and Stephen R. Meech[1]*

*1. School of Chemistry, University of East Anglia, Norwich NR4 7TJ, U.K; 2. Department of Chemistry, Stony Brook University, Stony Brook, New York 11794, United States; 3 Central Laser Facility, Research Complex at Harwell, Rutherford Appleton Laboratory, Didcot OX11 0QX, U.K. 4. Department of Biophysics, Medical School, University of Pecs, 7624 Pecs, Hungary*

## Contents

**1. Experimental methods section** .......... 2

**a.) Steady-State Absorption and Emission:** .......... 2

**b.) Fluorescence up-conversion:** .......... 2

**c.) Visible transient absorption (TA):** .......... 3

**d.) Time-resolved Infra-red spectroscopy (TRIR):** .......... 3

**2. Cloning, Expression and Purification of Kohinoor and Padron0.9** .......... 3

**3. Experimental Data** .......... 4

Figure S1. Plot of interactions between the on-state chromophore and surrounding amino-acid residues of a) Kohinoor and b) Padron0.9 (generated from LIGPLOT). The interaction map of Padron0.9 shows a water molecule interacting with phenol ring of the chromophore through H-bonding interactions, which is absent in Kohinoor. It also shows the R66 residue is H- bonded with imidazolinone ring, giving a downshifted bleach at around 1660 $cm^{-1}$ in Padron0.9, an interaction absent in Kohinoor. .......... 5

Figure S2. Upconverted fluorescence decay traces of Padron0.9, Koh-S176F and Kohinoor in $D_2O$ showing rising components in Padron0.9 and Koh-S176F but absent in Kohinoor. The grey trace shows the IRF of ~80 fs. .......... 5

TableS1: Time constants (τ) and amplitudes (α) obtained by fitting the time-resolved fluorescence (TRF) decay traces using a sum of two exponential model. Negative amplitudes correspond to a rising component obtained in Padron09 and Koh-S176F. .......... 6

Figure S3. Transient absorption spectra of (a) Padron0.9, (b) Koh-S176F, and (c) Padron2 following 405 nm excitation of the on-state neutral cis chromophore in $H_2O$. (d, e, and f) EADS obtained from global analysis of the TA data using a sequential model consisting of three exponential decay components and a fixed long constant. .......... 7

Figure S4. Transient absorption spectra of Padron0.9(R66K) in $D_2O$ following 405 nm excitation of the on-state neutral cis chromophore. .......... 7

Figure S5. Kinetic traces shown at specific wavelengths obtained by global fitting of the TA data of a) Padron0.9, b) Koh-S176F, and c) Padron2 in D2O. .......... 8

Figure S6. DADS obtained from global analysis of the TA data of a. Padron0.9, b. Koh-S176F and c. Padron2 using a parallel model consisting of multistep decay components and a fixed long constant. ....8

Figure S7. Kinetic traces shown at specific wavelengths obtained by global fitting of the TA data of a) Padron0.9, b) Koh-S176F, and c) Padron2 in $H_2O$. ....9

Figure S8. Overlay of the crystal structures of Padron0.9 (pink) and Kohinoor (green) showing R66 is displaced away from the chromophore carbonyl, whereas in Padron0.9 it lies closer and forms a H-bond with the carbonyl. ....9

Figure S9. a) TA data of Kohinoor following 405 nm excitation of the on state in $H_2O$shown at specific pump-probe delays, showing monotonic decay of the neutral SE and TA signals. b) TRIR spectra recorded after excitation of the ON-state at 380 nm in $D_2O$. The aterisks mark bleach modes related to the isolated chromophore....10

Figure S10. TRIR spectra of Padron0.9(R66K) mutant recorded after excitation of the ON-state at 380 nm in $D_2O$. ....11

Figure S11. Kinetic traces shown at specific wavelengths obtained by global fitting of the TRIR data of a) Padron0.9, b) Koh-S176F, and c) Padron2 in $D_2O$. ....12

Figure S12. DADS obtained from global analysis of the TRIR data of a. Padron0.9, b. Koh-S176F and c. Padron2 using a parallel model consisting of multistep decay components and a fixed long constant. ....12

## 1. Experimental methods section

**a.) Steady-State Absorption and Emission:** The UV–Visible ground-state absorption spectra were recorded using a Perkin Elmer Lambda XLS spectrophotometer in quartz cuvettes of 1 cm optical path length. Room temperature emission spectra were recorded using an Edinburgh Instruments FS5 spectrofluorometer under right angle configuration. The sample absorbance was kept below 0.1 (<2 μM), to minimize reabsorption effects, and excitation and emission bandwidths of 2 nm.

**b.) Fluorescence up-conversion:** The ultrafast fluorescence up-conversion setup has been described in detail elsewhere[1]. The experiment uses a femtosecond oscillator (Coherent Micra-10), a mode-locked Ti:Sapphire Laser producing pulses centred at 800 nm with a duration of ca. 20 fs, a repetition rate of 76 MHz, and a power output of around 750 mW. Excitation is achieved using the second harmonic at ca. 400 nm and the average power at the sample of up to 9 mW. For protein samples, the power was reduced to 3 mW to minimize photodamage. Upconversion was carried out in a 300-micron BBO crystal, providing an instrument response time of 75 fs, as determined by up-conversion of solvent Raman scattering. To maintain the samples in their original ON- or OFF-states, the reservoir was continuously illuminated with 505 nm LED to retain the ON state.

**c.) Visible transient absorption (TA):** The transient absorption setup used here has been detailed elsewhere2. The pump and probe beams were generated using an 800 nm fundamental output beam produced by a Spectra Physics-Mai Tai Laser oscillator and amplified by a Ti:Sapphire regenerative amplifier (Spectra Physics-Spitfire ACE). This amplified output pulse, characterized by a duration of 100 fs at 800 nm, a repetition rate of 1 kHz, and an energy of 5 mJ per pulse, served to drive the optical parametric amplifier (OPA, Light Conversion TOPAS Prime), thereby producing a tuneable pump pulse for sample excitation. A white light continuum (WLC) probe was generated by focusing part of the fundamental 800 nm beam onto a 3 mm thick CaF2 window, yielding a broadband continuum ranging from 350 to 650 nm. Transient absorption spectra presented in this work were recorded following sample excitation at 405 nm. The energy of the pump beam at the sample cell was attenuated to 300 μW (300 nJ). All measurements were conducted in 1 mm flow cuvettes with OD <1 (<concentration 50 μM). To maintain the samples in their original ON- or OFF-states, the reservoir was continuously illuminated with 405 nm or 505 nm LEDs, respectively.

**d.) Time-resolved Infra-red spectroscopy (TRIR):** Time-resolved infrared (TRIR) spectroscopy was conducted using the ULTRA or LifeTime systems at the Central Laser Facility within the Research Complex at Harwell[3]. Transmitted light from the sample was measured with pump-on and pump-off, employing a visible pulse to excite the sample, followed by an infrared probe to capture the vibrational spectrum. The sample was excited with 405 nm light pulses (300 nJ) in a 150 micron spot size at a repetition rate of 1 kHz, yielding high signal-to-noise spectra with approximately 200 fs resolution. Data were recorded at magic angle polarization conditions. Protein samples, prepared at a concentration of 1–2 mM in D2O, were analyzed using a 50 µm path length CaF2 cell. To reduce photobleaching and sample degradation, the sample cell was rastered while the samples were flowed through the cell at a rate of approximately 1.5 mL/min. The spectra were calibrated against the IR spectrum of polystyrene. To maintain the samples in their original ON- or OFF-states, the reservoir was continuously illuminated with 405 nm or 505 nm LEDs, respectively.

## 2. Cloning, Expression and Purification of Kohinoor and Padron0.9

The pRSETb plasmid that encoded the gene of Kohinoor (1-225) from Takeharu Nagai (Addgene plasmid # 67770) served as a template for site-directed mutagenesis. The Kohinoor S176F variant was generated using QuickChange mutagenesis and KOD Xtreme HotStart polymerase (Novagen). The codon-optimized gene encoded Padron0.9 (1-225) was synthesized and cloned into the pET15b vector in frame with an N-terminal six-His tag using the NdeI and BamHI restriction sites (Genscript). The pBAD plasmid encoding the gene of Padron2.0 (1-230) in frame with an N-terminal six-His Tag was a gift from Stefan Jakobs (Addgene plasmid #172355). All the final constructs were confirmed by Sanger Sequencing. The *E.Coli* Bl21 (DE3) competent cells were transformed with the pRSETb Kohinoor S156F, pET15b Padron0.9, or pBAD Padron2.0 plasmid and plated on an LB-Agar plate containing 100 $\mu$g/mL of ampicillin. The overnight culture was prepared by inoculating LB Miller broth, containing 100 $\mu$g/mL of ampicillin, with a single colony and incubating at 37 °C in an orbital shaker (250 rpm). After overnight incubation at 37 °C, 10 mL of the overnight culture was inoculated into 1 L of 2x-YT media (Fisher Bioreagents, BP9743-5) with the appropriate antibiotic. The cultures were incubated at 37 °C in an orbital shaker (250 rpm) until the $OD_{600}$ ∼0.6-0.8 was 0.6- 0.8, and then, the temperature was lowered to 18 °C. Once the $OD_{600}$ was ~0.8-1, 1 mM isopropyl β-D-1-thiogalactopyranoside (IPTG, Gold Biosciences) for Kohinoor S156F and Padron0.9, or 1 g of L-arabinose for Padron2.0 was added to induce protein expression. The cells were harvested after 16 h post-induction by centrifugation at 5,000 RPM (6,238 x g) (4 °C) for 20 min and the cell pellet was stored at −20 °C until needed

The cell pellet was resuspended in 40 ml of lysis buffer (20 mM Tris pH 8, 150 mM NaCl, buffer A) and lysed by sonication. The cell debris was removed by ultracentrifugation at 40,000 rpm (185,511 x g) for 1 h (4 °C) and filtered by a 40μm constrainer (Wards Sciences). The supernatant was then loaded into a 5 ml Ni-NTA column (GE) and washed with 10-20 CV of buffer A containing 10-30 mM of Imidazole. The protein was eluted with 500 mM Imidazole. Fractions containing protein were pooled and concentrated by a 10 kDa cut-off concentrator and desalted using the HiPrep 26/10 desalting column (GE) with buffer A. (AKTA FPLC purifier). Pure protein fractions were collected, and the purity of the protein was confirmed by SDS-PAGE. The concentration of the proteins were determined by the absorbance spectrum using the extinction coefficient (Kohinoor $\varepsilon_{495}$ = 62,900 $M^{-1} cm^{-1}$ Padron0.9 $\varepsilon_{495}$ = 43,000 $M^{-1}$)[5]. The protein was lyophilized and resuspended in $H_2O$ for TA and TRF measurements or D2O for the TRIR and TRMPS measurements.

**Mutagenesis Primers**

S176F_Forward:

5’—CAC TAC CGA TGT GAC TTC AAA ACT ACT TAT AAA GC—3’

S176F_Reverse:

5’—GC TTT ATA AGT AGT TTT GAA GTC ACA TCG GTA GTG—3’

## 3. Experimental Data

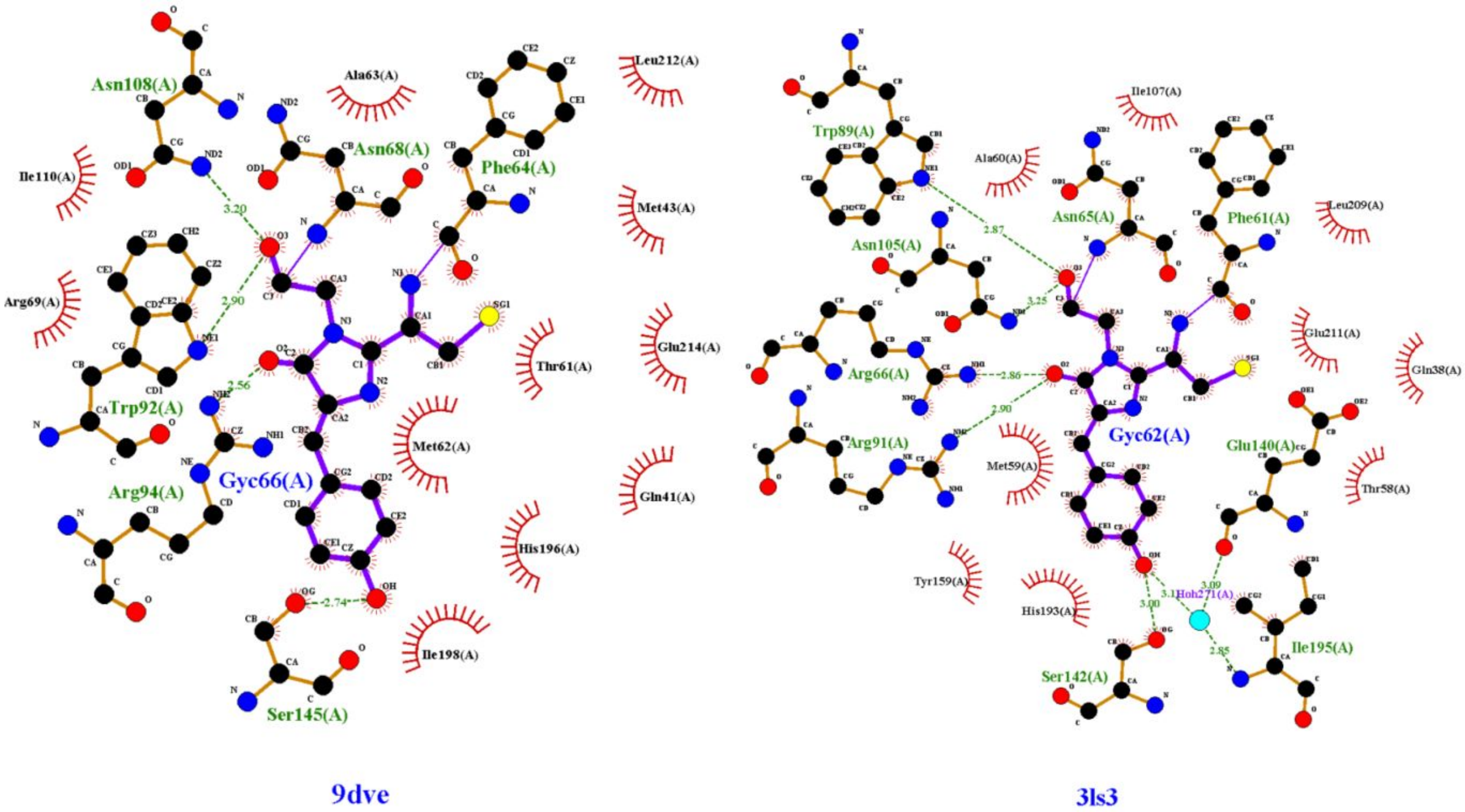

Figure S1. Plot of interactions between the on-state chromophore and surrounding amino-acid residues of a) Kohinoor and b) Padron0.9 (generated from LIGPLOT). The interaction map of Padron0.9 shows a water molecule interacting with phenol ring of the chromophore through H-bonding interactions, which is absent in Kohinoor. It also shows the R66 residue is H- bonded with imidazolinone ring, giving a downshifted bleach at around 1660 $cm^{-1}$ in Padron0.9, an interaction absent in Kohinoor.

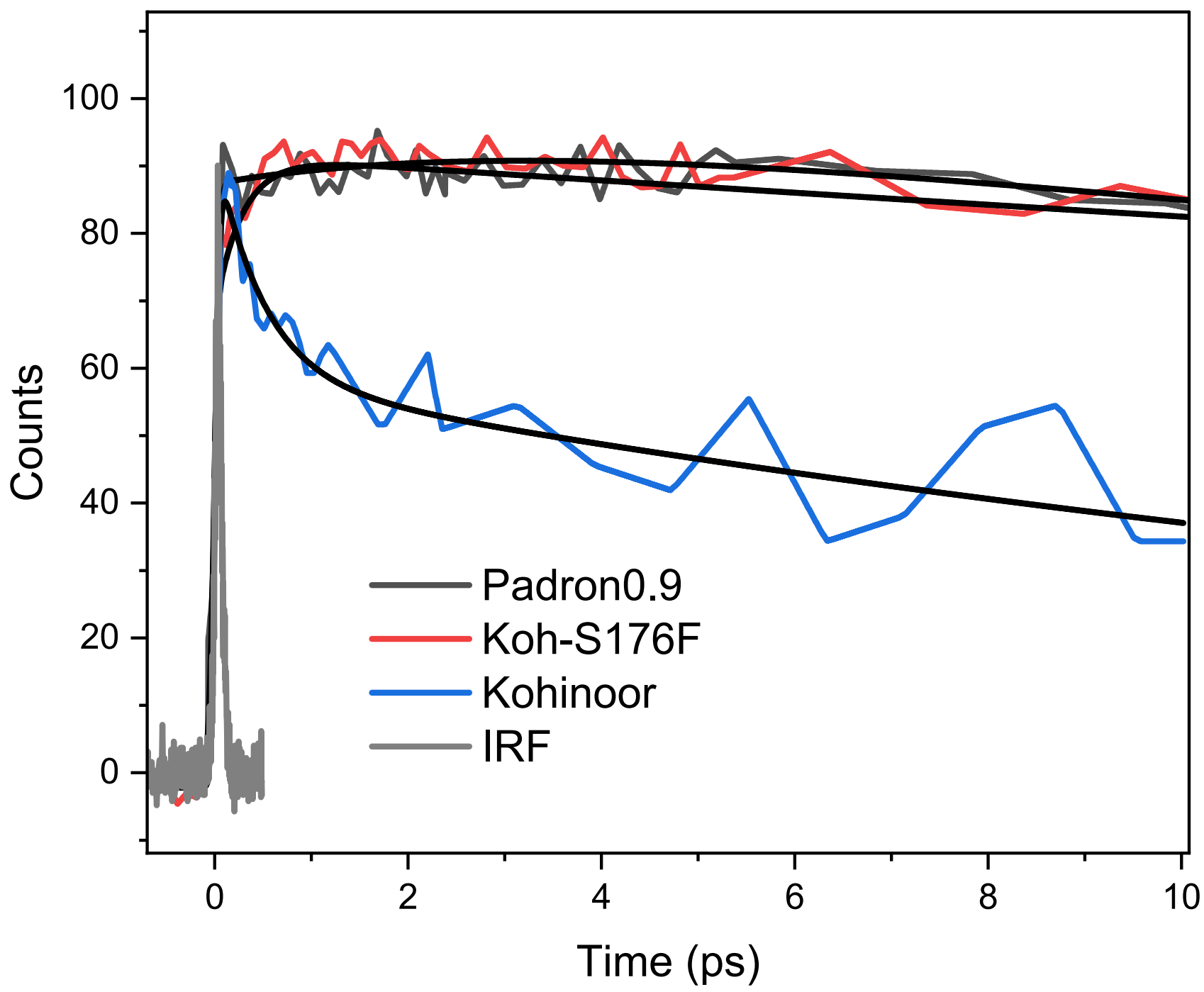


Figure S2. Upconverted fluorescence decay traces of Padron0.9, Koh-S176F and Kohinoor in $D_2O$ showing rising components in Padron0.9 and Koh-S176F but absent in Kohinoor. The grey trace shows the IRF of ~80 fs.

TableS1: Time constants (τ) and amplitudes (α) obtained by fitting the time-resolved fluorescence (TRF) decay traces using a sum of two exponential model (estimated errors of ±20%). Negative amplitudes correspond to a rising component obtained in Padron09 and Koh-S176F.

| $H_2O$ | $\alpha_1$ | $\tau_1$ (ps) | $\alpha_2$ | $\tau_2$ (ps) |
|---|---|---|---|---|
| **Padron0.9** | -36 | 2 | 119 | 76 |
| **Koh-S176F** | -6 | 0.28 | 25 | 97 |
| **Kohinoor** | 16 | 0.4 | 20 | 22 |
| $D_2O$ | | | | |
| **Padron0.9** | -12 | 3.6 | 88 | 57 |
| **Koh-S176F** | -8 | 2.8 | 24 | 60 |
| **Kohinoor** | 22 | 0.4 | 8 | 27 |

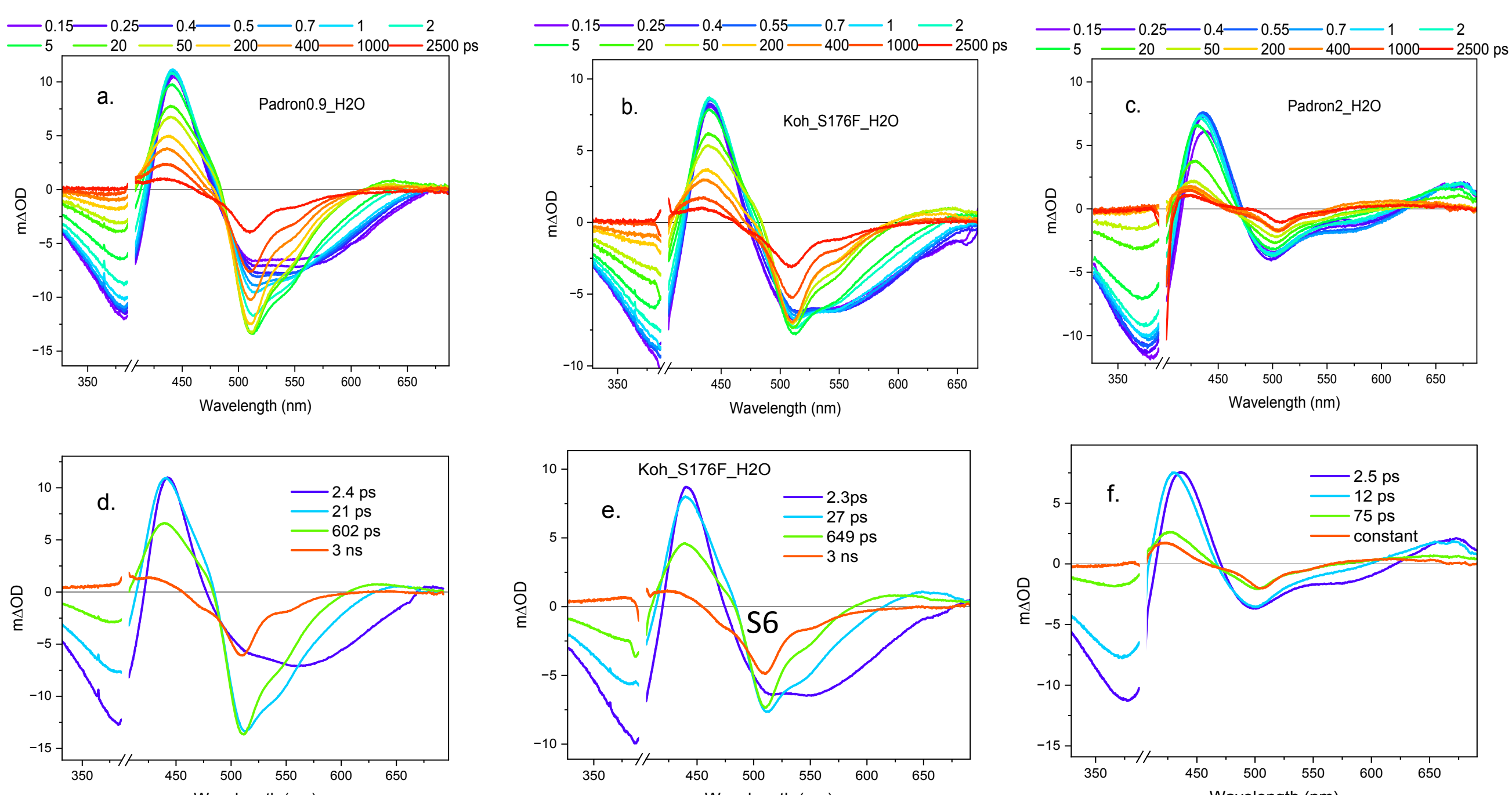

Figure S3. Transient absorption spectra of (a) Padron0.9, (b) Koh-S176F, and (c) Padron2 following 405 nm excitation of the on-state neutral cis chromophore in $H_2O$. (d, e, and f) EADS obtained from global analysis of the TA data using a sequential model consisting of three exponential decay components and a fixed long constant.

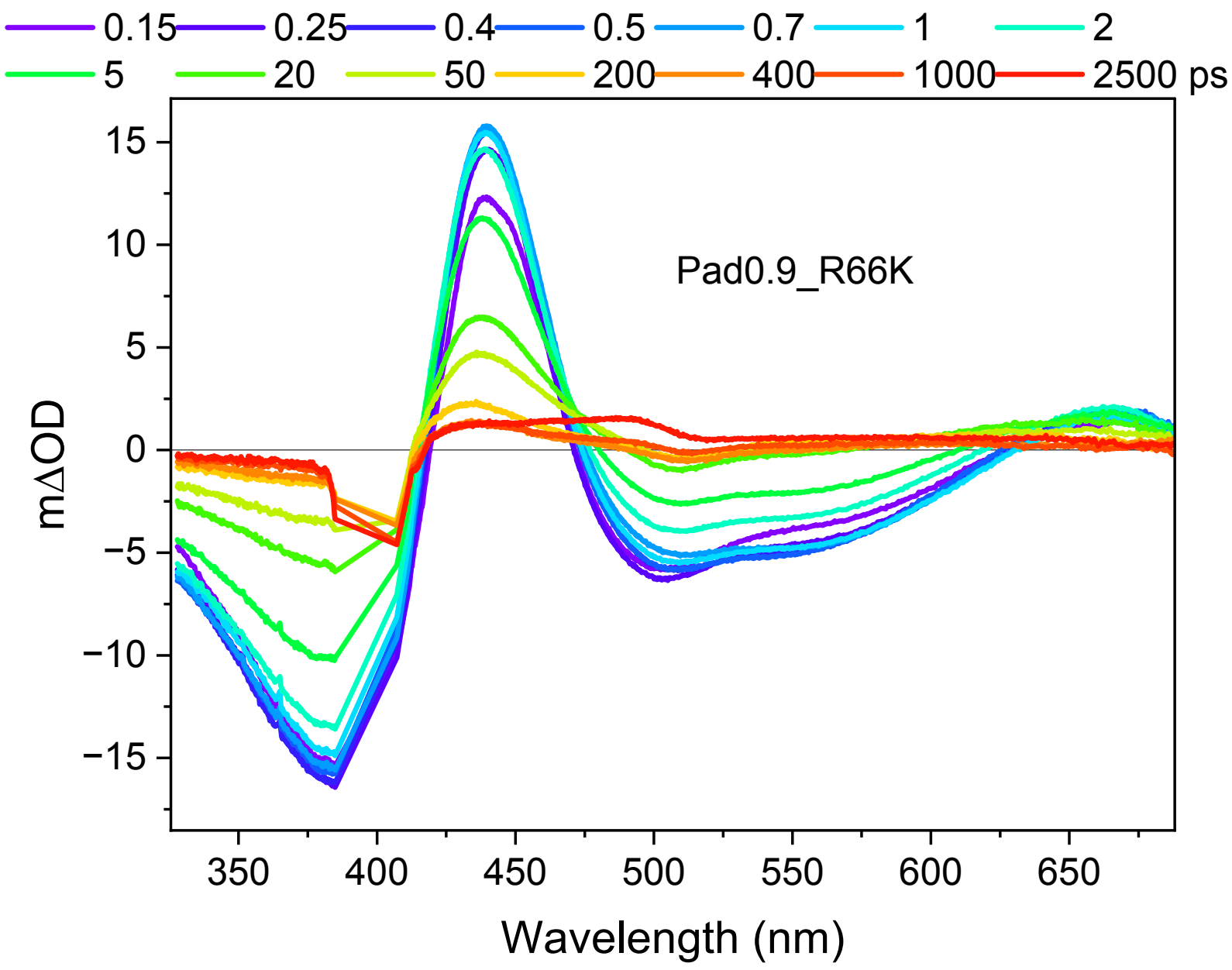


Figure S4. Transient absorption spectra of Padron0.9(R66K) in $D_2O$ following 405 nm excitation of the on-state neutral cis chromophore.

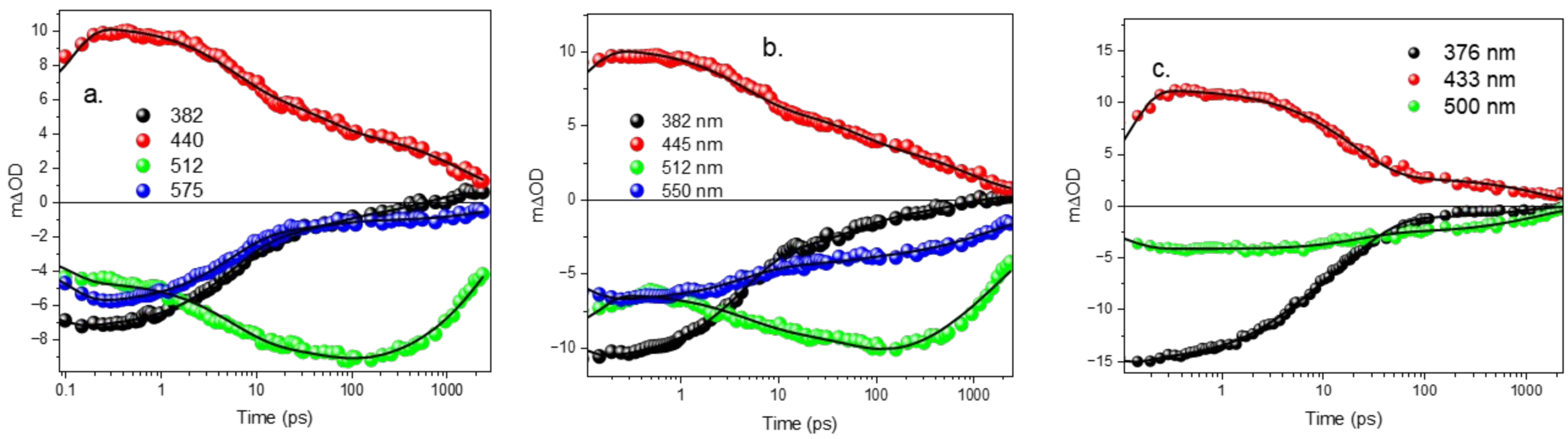


Figure S5. Kinetic traces shown at specific wavelengths obtained by global fitting of the TA data of a) Padron0.9, b) Koh-S176F, and c) Padron2 in D2O.

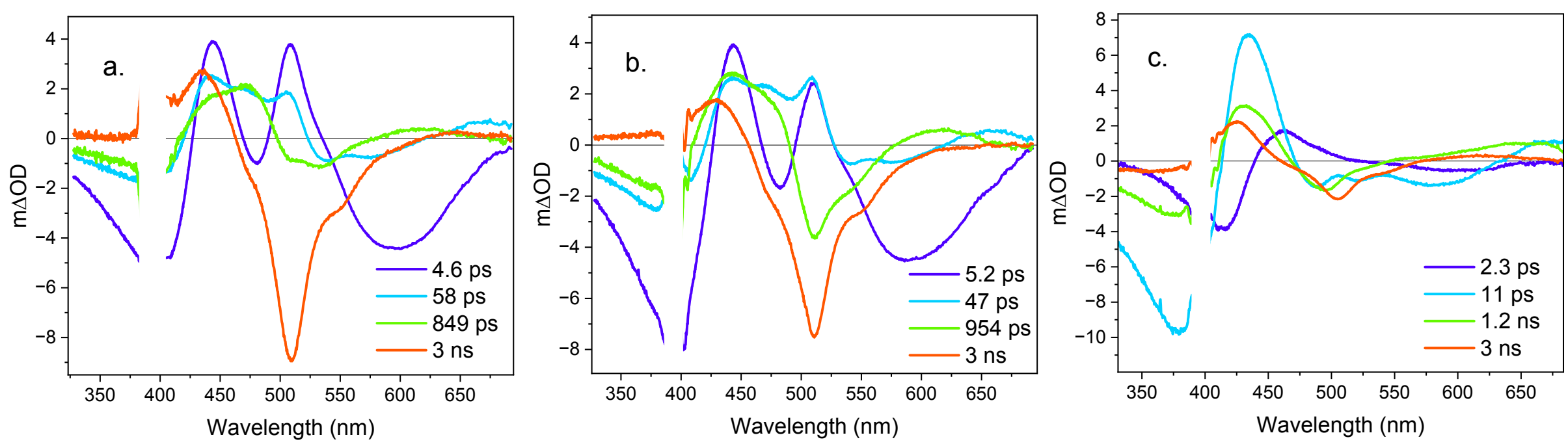


Figure S6. DADS obtained from global analysis of the TA data of a. Padron0.9, b. Koh-S176F and c. Padron2 using a parallel model consisting of multistep decay

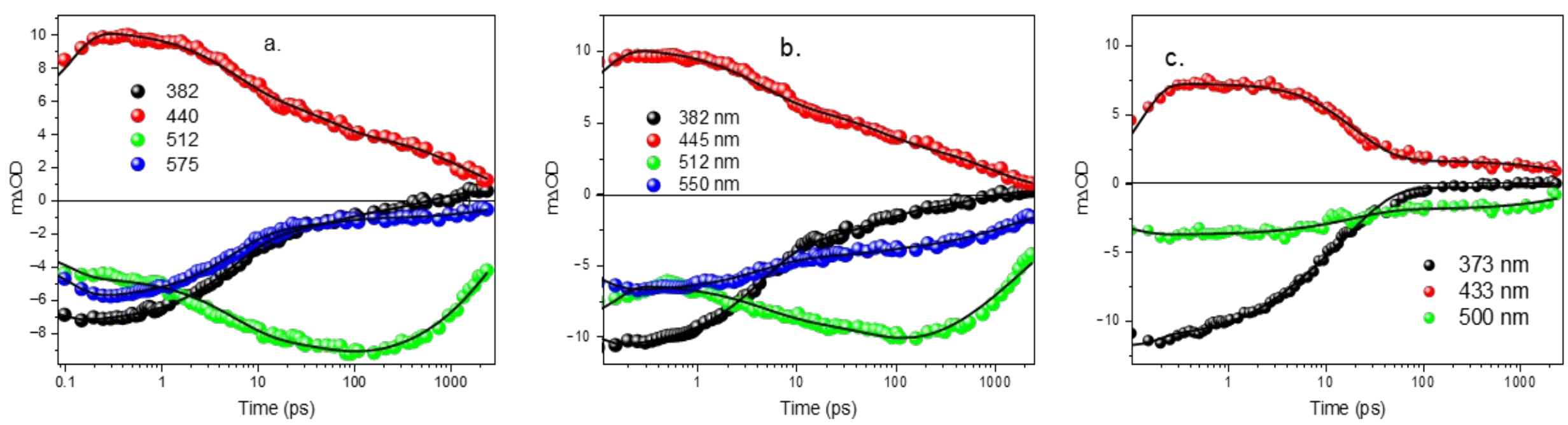


components and a fixed long constant.

Figure S7. Kinetic traces shown at specific wavelengths obtained by global fitting of the TA data of a) Padron0.9, b) Koh-S176F, and c) Padron2 in $H_2O$.

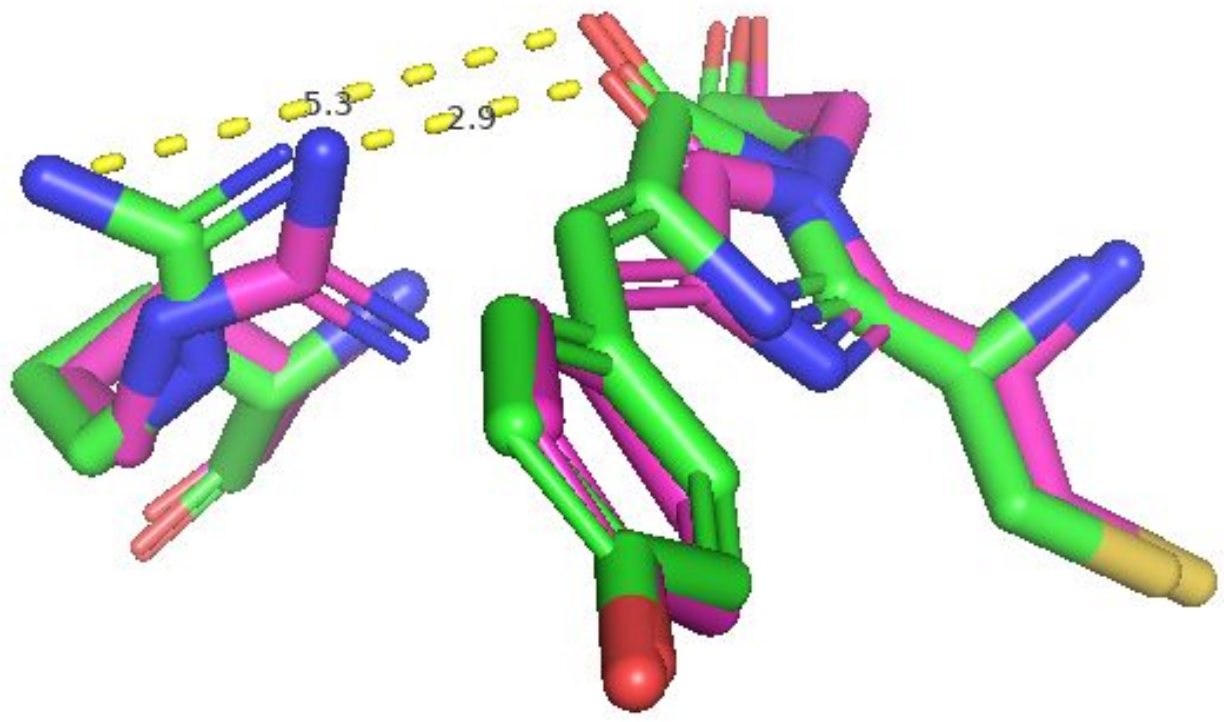


Figure S8. Overlay of the crystal structures of Padron0.9 (pink) and Kohinoor (green) showing R66 is displaced away from the chromophore carbonyl, whereas in Padron0.9 it lies closer and forms a H-bond with the carbonyl.

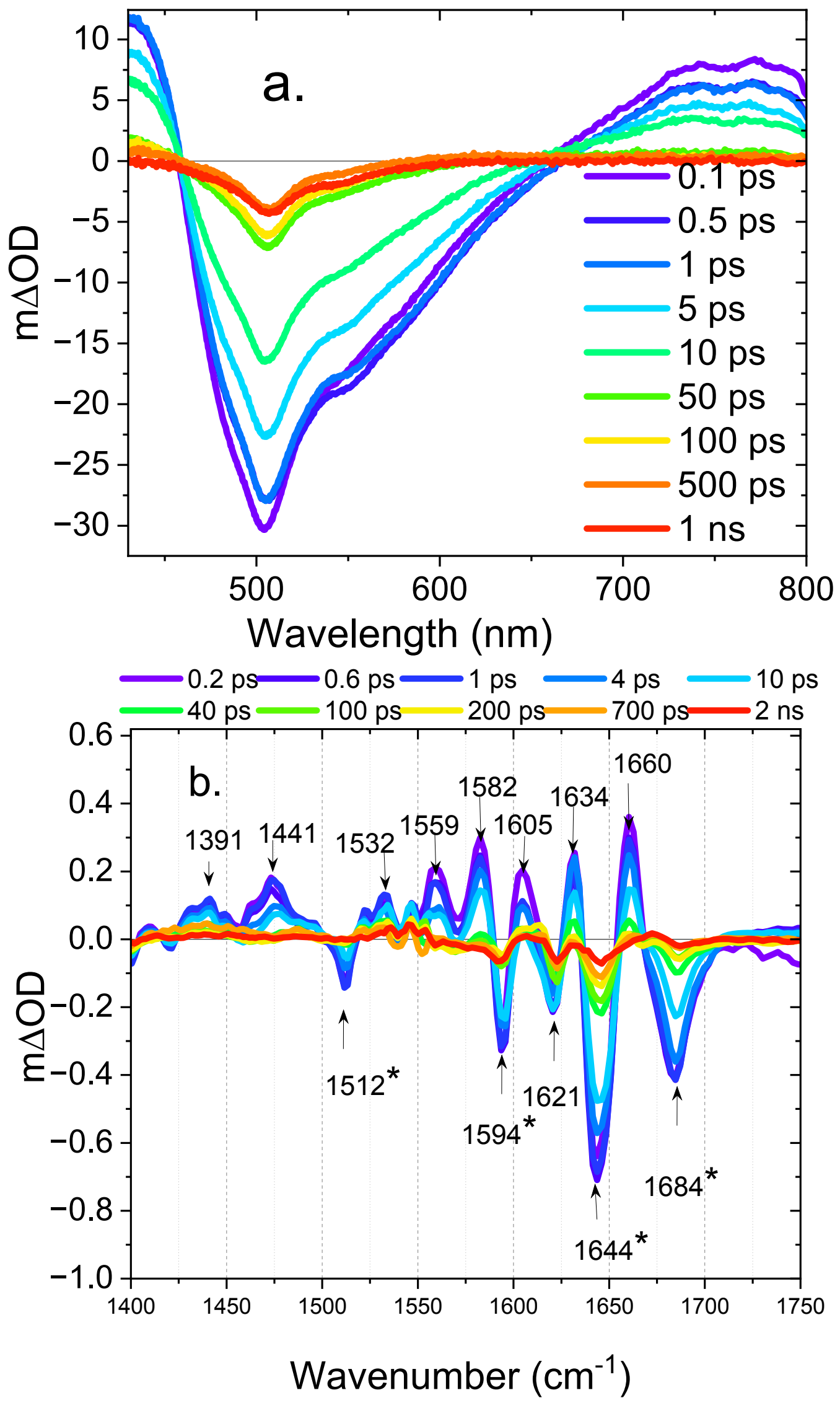


Figure S9. a) TA data of Kohinoor following 405 nm excitation of the on state in $H_2O$shown at specific pump-probe delays, showing monotonic decay of the neutral SE and TA signals. b) TRIR spectra recorded after excitation of the ON-state at 380 nm in $D_2O$. The aterisks mark bleach modes related to the isolated chromophore.

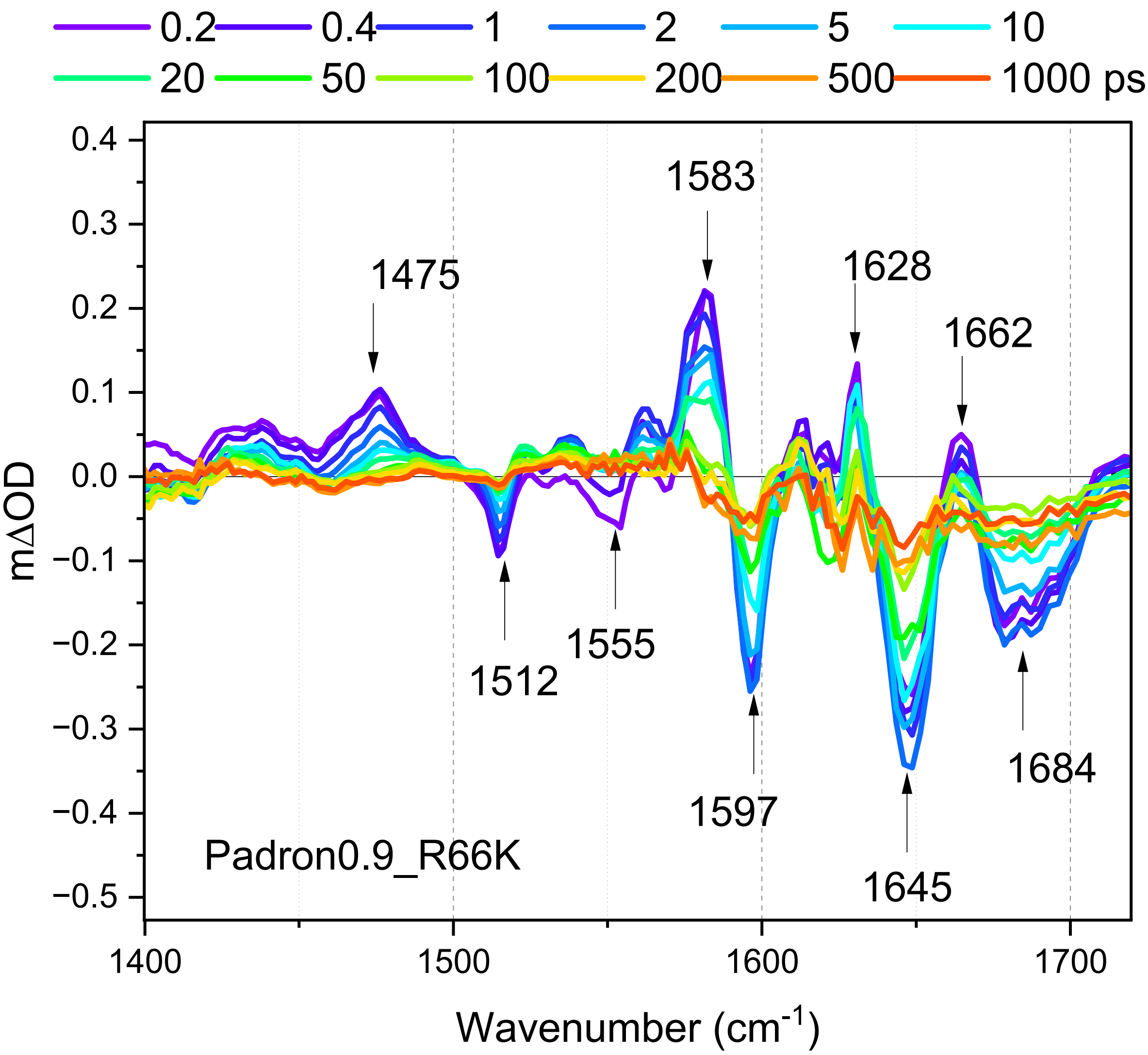


Figure S10. TRIR spectra of Padron0.9(R66K) mutant recorded after excitation of the ON-state at 380 nm in $D_2O$.

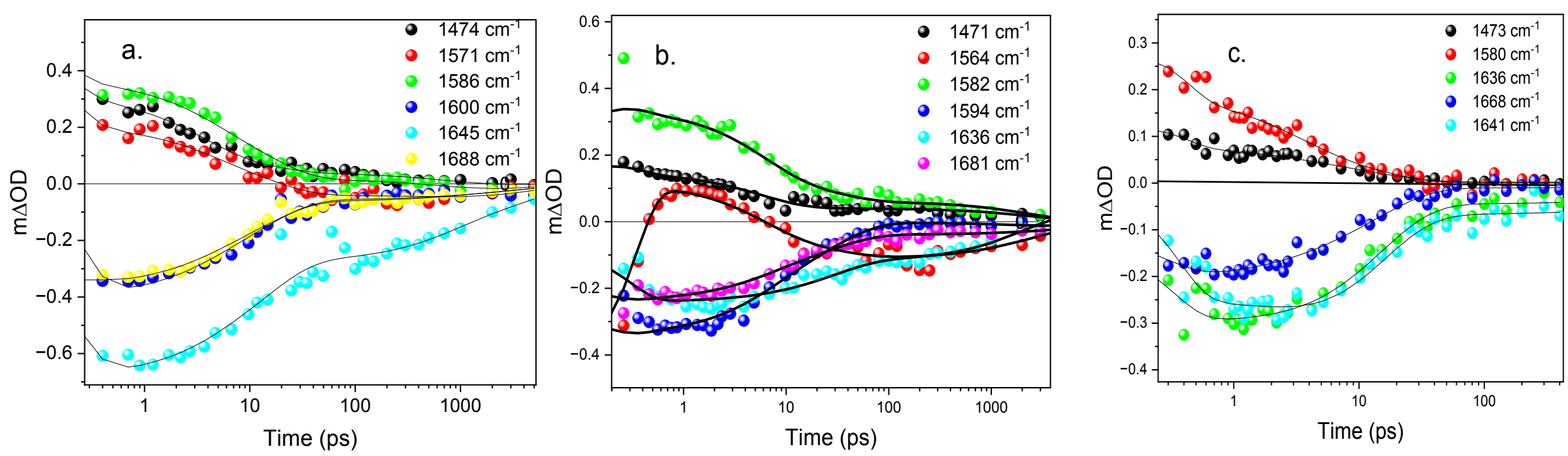

Figure S11. Kinetic traces shown at specific wavelengths obtained by global fitting of the TRIR data of a) Padron0.9, b) Koh-S176F, and c) Padron2 in $D_2O$.

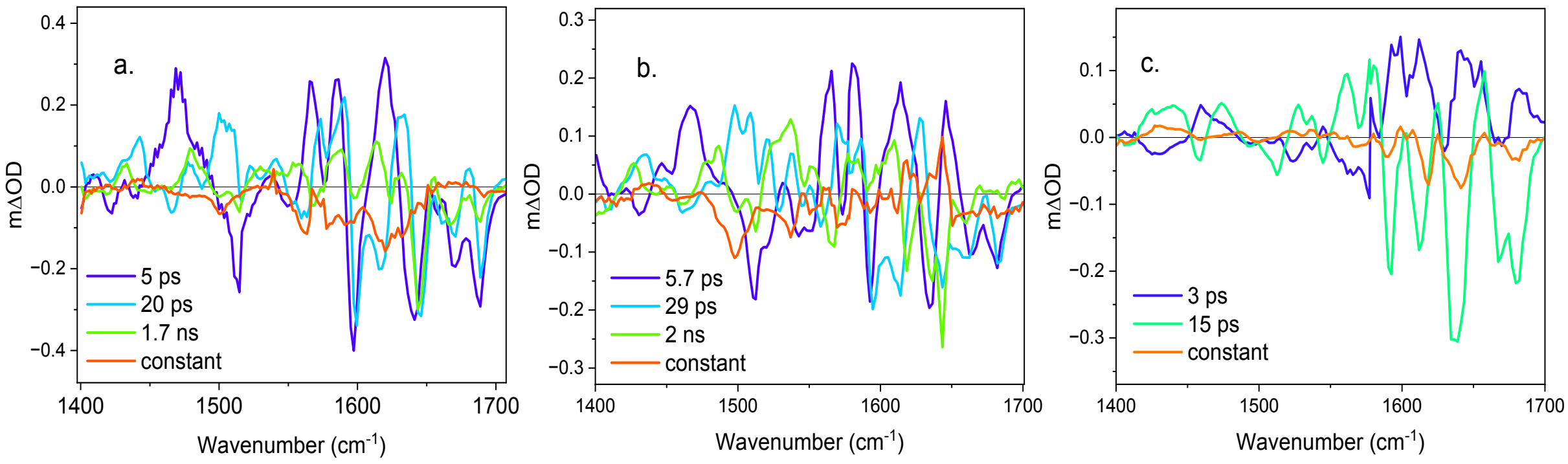


Figure S12. DADS obtained from global analysis of the TRIR data of a. Padron0.9, b. Koh-S176F and c. Padron2 using a parallel model consisting of multistep decay components and a fixed long constant.
